# Combined inoculation of rhizobacteria with *Mesorhizobium* promotes growth, nutrient contents, and protects chickpea against *Fusarium redolens*

**DOI:** 10.3934/microbiol.2025015

**Published:** 2025-04-14

**Authors:** Sabrine Balti, Yassine Mabrouk, Mouna Souihi, Imen Hemissi, Ismail Amri, Ethan Humm, Noor Khan, Ann M. Hirsch

**Affiliations:** 1 Laboratory of Biotechnology and Nuclear Technology (LR16CNSTN01), National Centre for Nuclear Sciences and Technologies (CNSTN), Sidi Thabet Technopark, 2020, Tunisia; 2 Faculty of Sciences of Bizerte, Carthage University, 7021 Jarzouna, Tunisia; 3 National Institute of Agronomic Research of Tunis, Hédi Karray Street, 2049 Tunis, Tunisia; 4 Department of Molecular, Cell, and Developmental Biology and Molecular Biology Institute, University of California, Los Angeles, CA 90095, USA

**Keywords:** Chickpea, PGPR, plant growth, biological control, *Fusarium redolens*

## Abstract

Chickpea (*Cicer arietinum* L.) is considered a cheap source of plant protein. In Mediterranean regions, and particularly in Tunisia, fungal attacks are likely to further aggravate drought stress and increase the economic vulnerability of chickpea production. Plant growth-promoting rhizobacteria (PGPR) and rhizobia have the potential to enhance plant growth and mitigate the adverse effects of biotic and abiotic stresses. The objective of this study was to isolate non-rhizobial rhizosphere bacteria from the soil and evaluate their ability to enhance plants' growth and symbiotic performance and to control chickpea wilt caused by *F. redolens*. A total of 26 bacterial isolates from rhizosphere soil samples were subsequently evaluated for their antagonistic properties against five phytopathogenic fungi (*Fusarium oxysporum solani, Fusarium oxysporum matthioli*, *Fusarium oxysporum MN-2*, *Fusarium oxysporum* 184, and *Fusarium rdolens*). Seven bacterial isolates demonstrated *in vitro* plant-beneficial characteristics and/or antagonistic activity against 5 *Fusarium* strains. Two bacterial strains including *Streptomyces diastaticus* subsp. *diastaticus* and *Bacillus subtilis* were chosen for additional investigation because they showed the greatest number of plant growth-promoting (PGP) traits and exhibited an antagonistic effect on pathogens. Assays conducted in pots showed that PGPRs co-inoculated with *Mesorhizobium* sp. Bj1 protected chickpea plants from *F. redolens* infection and enhanced plant growth and nutrient uptake. Pot experiments carried out in a greenhouse further demonstrated that the co-inoculation of chickpea plants with the bacterial strains and a *Mesorhizobium* strain lessened the severity of the *F. redolens* infection. These results suggest that co-inoculation with *S. diastaticus* subsp. *diastaticus* and *Mesorhizobium* sp. Bj1 may act as a helpful bioformulation to boost chickpea plants' growth and protect them from wilting. Other PGPR candidates included *Mesorhizobium* spp. and *B. subtilis* strains. Both *Mesorhizobium* sp. Bj1 and the uninoculated plants were used as controls. The association of PGPR with other inoculants potentially could substitute for chemical fertilizers, and testing of PGPR under field conditions will further elucidate their effectiveness on grain yields of chickpea.

## 1. Introduction

Chickpea (*Cicer arietinum* L.) ranks among the most widely cultivated vegetables across various regions globally. In Tunisia, however, both the area dedicated to its cultivation and the overall production have experienced considerable fluctuations and declines, primarily due to biotic and abiotic factors impacting the chickpea crop [1]. The principal diseases that threaten chickpea include *Ascochyta rabiei*, *Fusarium oxysporum* f. sp. *ciceri*, *Botrytis cinerea*, and *Rhizoctonia solani* [2]. Recently, a study by Bouhadida et al. [3] revealed, for the first time, the existence of *Fusarium redolens* causing wilting on chickpea. Research carried out by Jimenez-Fernandez et al. [4] also demonstrated that the infection of chickpeas by *F. redolens* results in a disease syndrome akin to that produced by the yellowing pathotype of *F. oxysporum* f. sp. *Ciceris* (FOC). Morphological diagnosis does not readily distinguish *F. redolens* from FOC, as both species elicit comparable symptoms in chickpea plants.

The management of *Fusarium* wilt in chickpeas presents significant challenges, because no singular control strategy has proven to be entirely effective [5]. This disease is classified as monocyclic, with its progression primarily influenced by the pathogen's initial inoculum. Consequently, effective management should focus on both preventing the introduction of the pathogen and minimizing the quantity and/or effectiveness of the primary inoculum [6]. Effective strategies for pathogen control are urgently needed for these reasons.

In agriculture, the most widely used methods for the management of fungal diseases are chemical-based fungicides. However, in addition to their adverse health and environmental effects, the excessive use of chemicals is responsible for the emergence of pathogens with multiple resistances to fungicides [7]. Research is currently focused on antagonism and biological control agents for plant diseases as viable alternatives to synthetic pesticides, owing to their perceived safety and reduced environmental impact. This approach has seen successful development over the past decade. It relies on diminishing inoculant or pathogenic activity through the natural presence of one or more organisms, achieved by managing the environment, the host, or the antagonists.

Numerous bacterial strains, including *Bacillus*, *Pseudomonas*, and, more recently, the *Rhizobium* group, have been isolated and demonstrated efficacy in managing various soilborne plant pathogenic fungi in both greenhouse and field conditions [8]–[10]. This approach relies on the use of several antagonistic microorganisms such as plant growth-promoting rhizobacteria (PGPR), which are used to control fungal diseases in chickpea. Indeed, PGPRs play the role of biofertilizers and directly improve plant growth due to their ability to fix nitrogen, solubilize phosphates, and produce phytohormones. In addition, PGPRs can be considered as biopesticides, and the indirect improvement of plant growth results from their ability to suppress soil pathogens through the production of hydrogen cyanide, siderophores, and antibiotics and through competition for nutrients [11],[12]. Moreover, PGPRs can synthesize elicitors of resistance induced in chickpea plants, which reduces the severity of diseases [13],[14].

To improve the advantageous impact of rhizobacteria, this study aimed to investigate the antagonistic properties of certain bacterial strains isolated from soil against *Fusarium redolens*, the causative agent of chickpea wilt, as well as the plant growth-promoting rhizobacteria (PGPR) characteristics of these strains *in vitro*. Additionally, we have sought to examine the effects of co-inoculating chickpea plants with rhizobacteria on plant growth, nutrient content, and disease suppression in a controlled greenhouse environment.

## 2. Materials and methods

### 2.1. Plant material

The seeds of the chickpea variety Rebha used in this work were provided by the Selected Seeds Society (SOSEM) J3J8+QJW, South, Beja, Tunisia.

### 2.2. Bacterial isolates

Some bacterial strains that were isolated from soil and soil samples were obtained from the rhizospheres of various plants (Table 1), and the isolation of bacterial strains was performed using the selective method outlined by Travers et al. [15]. Specifically, 1 g of each sample was suspended in 10 mL of sterile distilled water. Subsequently, 1 mL of each suspension was introduced into 10 mL of Luria–Bertani (LB) nutrient broth that was buffered with sodium acetate at a concentration of 0.25 M and a pH of 6.8. Individual colonies were subjected to repeated streak plating on the same medium until purified isolates were achieved. Twelve additional bacterial strains were obtained from the Hirsch laboratory, University of California, Los Angeles (UCLA).

The *Mezorhizobium* strain Bj1 used in co-inoculation in the biocontrol tests was provided by the Legume Program, Field Crop Laboratory, National Institute of Agronomic Research of Tunis.

Gram staining was used to identify the chosen strains morphologically after they were observed using an immersion optical microscope. Following that, 16S rDNA gene sequencing was used for molecular identification. Genomic DNA was extracted, quantified using NanoDrop spectrophotometry, and qualitatively examined on a 1% w/v agarose gel according to the method described by [23]. The 16S rDNA genes were amplified using the universal primers fD1 (AGAGTTTG ATCCTGGCTCAG) and rD1(AAGGAGGT GATCCAGCC). Following automated sequencing of the purified polymerase chain reaction products, the resulting sequences were compared with those on the NCBI using the Blast tool.

### 2.3. Fungal strains

Five fungal strains from the genus *Fusarium* were utilized in the experiments. Four of these strains, specifically *F. oxysporum solani*, *F. oxysporum matthioli*, *F. oxysporum MN-2*, and *F. oxysporum 184*, were sourced from UCLA. The fifth strain, *F. redolens*, was obtained from the laboratory of plant protection at the Tunisian National Institute of Agronomic Research. All fungal strains were preserved on potato dextrose agar (PDA, Sigma) at a temperature of 4 °C.

### 2.4. Assessment of the antagonistic properties of bacteria against Fusarium strains in vitro

A preliminary screening test was performed utilizing the agar disc method on PDA medium to identify isolates exhibiting antagonistic properties from a collection of bacterial isolates. A fungal disc, measuring 6 mm in diameter, was centrally positioned in a 90-mm Petri dish filled with PDA medium, while a streak of bacterial isolates was placed 2.5 cm away from the disc on the same dish. Antagonistic interactions were noted as either positive (+) or negative (−), indicating presence or absence, respectively. Bacterial isolates that demonstrated antagonism (+) towards the fungal strains were selected for a subsequent round of screening through the *in vitro* double culture assay. The inhibition of fungal growth was assessed seven days post-incubation at 24 °C, and the percentage of reduction in mycelial growth was calculated using the appropriate formula:

$$I=\frac{R1-R2}{R1}x100$$

where R1 represents the mycelial growth observed on the plate without bacteria, while R2 indicates the mycelial growth directed towards the bacterial streaks. Each experiment was conducted three times, with three repetitions for each trial.

### 2.5. In vitro evaluation of bacterial strains' PGP traits

#### 2.5.1. Cellulase detection

The qualitative assessment of cellulase was performed following the methodology outlined by Kasana and his collaborators [16]. This involved point inoculation of the strain onto a basic agar medium that utilized carboxymethylcellulose as the exclusive carbon source. After a 5-day incubation period at 30 °C, all plates were treated with a 1% (w/v) solution of Congo red for 15 minutes, followed by destaining with 1 M NaCl for an additional 15 minutes. The presence of cellulase was confirmed by observing a zone of degradation surrounding the bacterial colonies.

#### 2.5.2. Ammonia production

The estimation of ammonia production was conducted following the methodology outlined by Abdelwahed et al. [17]. In summary, bacterial isolates were cultured in a peptone water broth maintained at a temperature of 30 ± 2 °C for a duration of 7 days. Subsequently, the cultures underwent centrifugation, and the resulting bacterial supernatants were allocated into various wells at a volume of 50 µL per well. To each well, 100 µL of Nessler's reagent was added, and the microplate was incubated at room temperature for 10 minutes. Following this incubation, 55 µL of each reaction mixture was transferred to a second 96-well microplate, to which 330 µL of ultrapure water was added, achieving a final volume of 385 µL. The optical density was then measured at 450 nm, and the concentration of ammonia was determined using a standard ammonium sulfate solution.

#### 2.5.3. Indole-3-acetic-acid production

Indole-3-acetic acid (IAA) production was established following the methodology outlined by Bric et al. [18]. The isolates were cultured in LB medium at a temperature of 28 °C for a duration of two days, with positive colonies identified using the Salkowski reagent. For the purpose of quantitative analysis, liquid LB medium supplemented with 0.1 g/L of L-tryptophan was inoculated with the isolates and incubated for three days at 28 °C. The quantities of IAA were measured in accordance with the procedures described by Gravel et al. [19]. A calibration curve was generated utilizing IAA obtained from Sigma-Aldrich.

#### 2.5.4. Phosphate solubilization assay

The ability of the isolates to solubilize inorganic phosphate was evaluated using the National Botanical Research Institute's Phosphate growth (NBRIP) medium, which consists of the following per liter: 10 g of glucose, 0.1 g of (NH_4_)_2_SO_4_, 0.2 g of KCl, 0.25 g of MgSO_4_·7H_2_O, 5 g of MgCl_2_·6H_2_O, and 5 g of tri-calcium phosphate (TCP) [20].

For the quantitative assessment of phosphate solubilization in liquid NBRIP medium, 20 mL of NBRIP containing tricalcium phosphate (TCP) was inoculated with 100 µL of the standardized bacterial suspensions, achieving a final concentration of 10^7^ colony-forming units (CFU)/mL. These suspensions were derived from a 24-hour preculture in King B medium. The flasks were then incubated at 28 °C in the dark while being agitated at 180 rpm. After a 48-hour incubation period, phosphate solubilization was evaluated through colorimetric analysis of the culture supernatant at 600 nm, utilizing a UV–visible spectrophotometer (UV-3100PC, VWR), in accordance with the method established by Fiske and Subbarow [21]. The results were expressed as mg of released phosphorus per mL. A calibration curve was generated using KH_2_PO_4_, and the variation in pH was determined by measuring the pH under the initial conditions and after 48 hours.

#### 2.5.5. Nitrogen fixation

To quantify nitrogen fixation, bacterial isolates were cultured on a nitrogen-free medium (NFM) for a duration of seven days, after which, the growth of the bacteria was assessed qualitatively [22].

#### 2.5.6. Siderophore assay

The quantification of siderophores was conducted using the Chrome Azurol S (CAS) assay. To prepare the CAS solution, 72.9 mg of hexadecyl trimethylammonium bromide (HDTMA) was dissolved in 40 mL of deionized water, while 60.5 mg of chromium azurol S was dissolved in 50 mL of deionized water. These solutions were then combined, and 10 mL of an Fe^3+^ solution (1 mM FeCl_3_ in 10 mM HCl) was added. The CAS solution was subsequently sterilized and mixed with autoclaved King's medium in a 1:9 ratio. For the quantification of siderophores, equal volumes (100 µL) of bacterial culture supernatants and CAS reagents were mixed. The mixture was incubated at room temperature for 20 minutes, after which, the absorbance was measured at 630 nm using a microplate reader. The quantities of siderophores were expressed as a percentage of siderophore units (psu) in accordance with Payne's method [23].

### 2.6. Greenhouse experiments

#### 2.6.1. Effect on plant growth parameters and nutrients contents

Seeds of the chickpea cultivar Rebha underwent surface sterilization using a 2% calcium hypochlorite solution for a duration of 30 minutes, followed by rinsing with sterile distilled water. The sterilized seeds were then immersed in a bacterial inoculum containing 10^8^ cells mL^−1^ of each bacterial strain before being transplanted into plastic pots filled with sterilized soil. For inoculum preparation, selected potential PGPRs were cultured in LB medium at 30 °C, and *Mesorhizobium* strains were cultured in Yeast Extract Mannitol (YEM) medium at 28 °C for 72 h. The cells obtained were adjusted to a final concentration of 10^8^ CFU/mL for each treatment.

The experiment consisted of five treatments as follows: T1 (Control), plants receiving no biofertilizer; T2, plants receiving the optimal dose of conventional N fertilizer; T3, plants inoculated only by bacterial Strain 1 (*Streptomyces diastaticus* subsp. *diastaticus*); T4, plants inoculated only by bacterial Strain 4 (*Bacillus subtilis)*; T5, plants receiving only *Mesorhizobium* strain Bj1 as the inoculum.

Plants were cultivated under a 16 h light cycle and a temperature of 25 °C. After 30 days of cultivation, measurements of the dry weight of both shoots and roots, and the lengths of shoots and roots were recorded. Shoot nitrogen concentration was measured according to the Kjeldahl method, whereas the phosphorus concentration was subsequently determined using the Olsen method [24].

#### 2.6.2. Assessment for biological control potential

To evaluate biological control potential, the two bacterial strains selected for their *in vitro* PGP traits. Strain 1 and Strain 4 were tested alone and in combination with the *Mesorhizobium* strain Bj1 to reduce the disease incidence of chickpea in pots under controlled conditions. First, these two bacterial strains and one *Mesorhizobium* strain were evaluated for compatibility with each other. Each PGPR isolate was cross-streaked with the *Mezorhizobium* strain on LB agar plates in a perpendicular direction and incubated in triplicate at 30 ± 1 °C for 48 h. The inhibition zone at the point of intersection was observed, and the results were marked as compatible for isolates having no inhibition zone at the point of intersection. Chickpea seeds were sterilized with 2% of sodium hypochlorite and rinsed six times with sterile distilled water. The seeds were then coated with the different bacterial suspensions (at 10^8^ cells per mL) and transferred to sterile soil at the rate of five seeds per pot. After 10 days, seedlings were inoculated with a fungal suspension consisting of 3 mL per seedling. For the preparation of the spore suspension, the fungus was grown on PDA at 25 °C, and Petri dishes of 2-week-old cultures were flooded with sterile water. Then the cultures were scraped to expel the spores. These spore suspensions were used for inoculation the same day after adjusting the concentration to 10^6^ spores/mL in distilled water. Four days after fungal inoculation, the seedlings were re-inoculated with the bacterial suspensions using 3 mL per seedling.

The experiment consisted of six treatments: T1 (Control), plants receiving no biofertilizer; T6, plants infected with the fungal strain *F. redolens*; T7, plants were inoculated only with bacterial Strain 1 (*Streptomyces diastaticus* subsp. *Diastaticus*) and infected by the fungal strain; T8, plants inoculated only by bacterial Strain 4 (*Bacillus subtilis*) and infected by the fungal strain; T9, plants infected by the fungus and co-inoculated with bacterial Strain 1 and *Mesorhizobium* strain Bj1; T10, plants infected by the fungus and co-inoculated with bacterial Strain 4 and *Mesorhizobium* strain Bj1.

After 30 days of fungal inoculation, the plant growth parameters were measured, and the severity of symptoms on individual plants was rated on a scale of 0 to 4, where 0 = 0%, 1 = 1–33, 2 = 34–66, 3 = 67–100%, and 4 = dead plant [25].

## 3. Data analysis

All data presented were expressed as the mean ± standard deviation (SD). An analysis of variance (ANOVA) was conducted for each dataset related to the disease using SPSS software (version 22). For independent repeated experiments, the data were assessed for significant differences. A *p*-value < 0.05 was deemed statistically significant.

## 4. Results

### 4.1. In vitro inhibition of hyphal growth by bacterial strains

**Figure 1.**
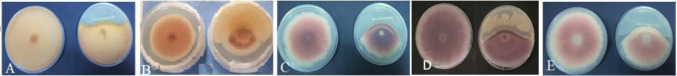
*In vitro* inhibition of mycelial growth of phyotpathogenic fungi: *Fusarium oxysporum solani* inhibited by Strain 1 (A), *Fusarium oxysporum MN-2* inhibited by Strain 4 (B); *Fusarium oxysporum matthioli* inhibited by Strain 1 (C); *Fusarium oxysporum 184* inhibited by Strain 4 (D); *Fusarium redolens* inhibited by Strain 1 (E) after 7 days of challenge.

A total of 26 bacterial isolates were derived from soil samples collected from the rhizosphere and subsequently evaluated for their antagonistic properties against five phytopathogenic fungi. Of these isolates, seven demonstrated antagonistic effects against the examined fungal strains, effectively inhibiting mycelial growth to varying degrees, contingent upon the specific fungal strain tested, at seven days post-inoculation (Table 1 and Figure 1). The five bacterial strains (S2, S3, S4, S6, and S7) successfully inhibited all the *Fusarium* strains assessed. Notably, Strain S7 exhibited the most significant inhibition, ranging from 76% to 79% on the seventh day following inoculation, while strains S1 and S5 resulted in the least growth inhibition against three to four fungal strains. An enhancement in the transparency of the fungus was noted in the plates that had been inoculated with several bacterial strains. In contrast, the uninoculated control exhibited a darker and more uniform appearance of the fungal colony across the Petri dish (Figure 1B).

**Table 1.** The effect of bacterial antagonists on the hyphal growth of fungal strains in the dual culture test.

Strain code	Bacterial strains	*Fusarium oxysporum solani*	*Fusarium oxysporum MN-2*	*Fusarium oxysporum matthioli*	*Fusarium oxysporum 184*	*Fusarium redolens*
Strain 1	*Streptomyces diastaticus* subsp. *diastaticus*	44,35 ± 18,50****	41,33 ± 2,30****	49,99 ± 3,33****	NE	49,49 ± 3,49****
Strain 2	*Bacillus* sp.	73,84 ± 10,66****	74,66 ± 6,11****	80 ± 0****	75,49 ± 8,60****	75,75 ± 0****
Strain 3	*Bacillus amyloliquefaciens*	67,17 ± 5,91****	74,66 ± 6,11****	79,99 ± 3,33****	72,55 ± 11,01****	75,75 ± 0****
Strain 4	*Bacillus subtilis*	69,39 ± 6,81****	73,33 ± 2,30****	81,11 ± 1,92****	73,99 ± 5,14****	79,79 ± 1,74****
Strain 5	*Bacillus* sp.	49,65 ± 6,36****	38,66 ± 2,30****	46,66 ± 0****	NE	NE
Strain 6	*Bacillus* sp.	73,84 ± 10,66****	74,66 ± 4,61****	80 ± 0****	75,18 ± 6,86****	76,76 ± 1,74****
Strain 7	*Bacillus* sp.	76,06 ± 12,58****	76 ± 4****	78,88 ± 1,92****	79,32 ± 4,88****	76,76 ± 1,74****

NE, no effect. The results are expressed as the mean percentages (±SD), and Dunnett's multiple comparison test was employed after two-way ANOVA to determine the significance relative to the control (0% of inhibition). * *p* < 0.05; ** *p* < 0.01; *** *p* < 0.001; **** *p* < 0.0001.

### 4.2. PGP characteristics of selected strains

**Figure 2.**
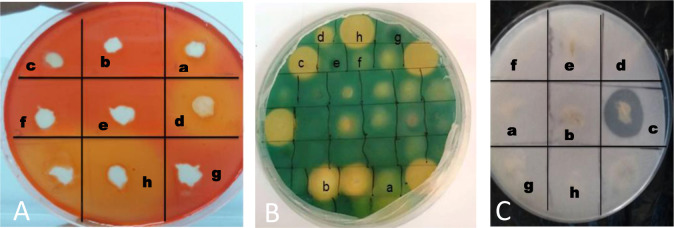
Cellulase production (A); siderophore production (B); inorganic phosphate solubilization (C). Strain 1 (a); Strain 2 (b); Strain 3 (c); Strain 4 (d); Strain 5 (f); Strain 6 (g); Strain 7 (h).

The chosen antagonists were additionally assessed for their potential to increase plant growth by producing iron-chelating agents and phytohormones *in vitro*. The *in vitro* PGP traits of the selected strains are summarized in Table 2. All of the selected strains were able to produce ammonia and siderophore, confirmed by the o-CAS assay through the appearance of halos around the colonies (Figure 2A). Most of the strains were able to produce IAA, to fix nitrogen, and to solubilize inorganic phosphate, which is essential for the PGP role of bacteria. S1 and S4 were positive for the most PGP traits and produced the highest amount of ammonia. In addition, these strains produced cellulase, which can degrade fungal cell walls (Figure 2).

**Table 2.** Bacterial strains' PGP activities *in vitro*.

Strain code	Bacterial strains	Cellulase	NH3 (mg/mL)	IAA (mg/mL)	Solubilization of inorganic phosphate (mg/mL)	Nitrogen fixation	Siderophore production
Strain 1	*Streptomyces diastaticus* subsp. *Diastaticus*	+	0.17 ± 0.01	0.01 ± 0.004	0.05 ± 0.004	+	++
Strain 2	*Bacillus* sp.	-	0.3 ± 0.07	0	0	+	+++
Strain 3	*Bacillus amyloliquefaciens*	-	0.39 ± 0.02	0.002 ± 0.001	0.15 ± 0.05	-	+++
Strain 4	*Bacillus subtilis*	+	0.69 ± 0.05	0.007 ± 0.001	0.04 ± 0.006	+	++
Strain 5	*Bacillus* sp.	-	0.18 ± 0.03	0.21 ± 0.004	0.14 ± 0.02	+	+
Strain 6	*Bacillus* sp.	-	0.11 ± 0.01	0	0	+	+
Strain 7	*Bacillus* sp.	+	0.25 ± 0.04	0	0.06 ± 0.01	-	+++

### 4.3. In planta evaluation of PGP traits

Rhizobacterial inoculation significantly improved the growth of the healthy chickpea plants and those infected by the fungus. Compared with control plants, chickpea treated with the bacterial strains showed a significant increase in shoot dry weight, with a greater effect seen when using *Streptomyces diastaticus* subsp. *distaticus* (Strain 1) (Table 3). In particular, the total dry biomass of the aerial parts of the plants inoculated with Strain 1 and Strain 4 and *Mesorhizobium* Bj1 bacteria was higher by approximately 10–17% than that of the control plants, while the shoot length was enhanced by 18–25% by the same strains. An increase was also observed for the length and dry weight of roots only for two bacterial strains S1 and S4.

**Table 3.** Effects of two bacterial strains (S1, *Streptomyces diasticus* subsp. *diasticus*; S4, *Bacillus subtilis*) and their combination with *Mesorhizobium* Bj1 on the growth of chickpea seedlings in pots.

	Treatments	Shoot length (cm)	Shoot dry weight (g)	Root length (cm)	Root dry weight (g)
T1	Chickpea	27.15 ± 4.25^b^	0.78 ± 0.09^a^	42.11 ± 5.24^b^	0.58 ± 0.08^ab^
T2	Chickpea + N	29.12 ± 4.57^c^	0.82 ± 0.1^ab^	44.15 ± 7.25^c^	0.62 ± 0.05^bc^
T3	Chickpea + S1	32.41 ± 5.21^d^	0.91 ± 0.11^b^	45.45 ± 6.21^cd^	0.68 ± 0.07^c^
T4	Chickpea + S4	34.51 ± 4.25^e^	0.85 ± 0.09^ab^	46.21 ± 5.21^d^	0.72 ± 0.09^c^
T5	Chickpea + Bj1	25.41 ± 3.21^a^	0.84 ± 0.08^ab^	40.11± 4.25^a^	0.56 ± 0.06^ab^

The values are the means (±SD) of 10 repetitions. Different letters indicate significance differences at the 5% level of probability using Duncan's multiple range tests.

The inoculation of chickpea plants with the bacterial strains allowed an increase in the growth parameters, and the biomasses obtained were better than those observed in the plants treated with nitrogen.

**Figure 3.**
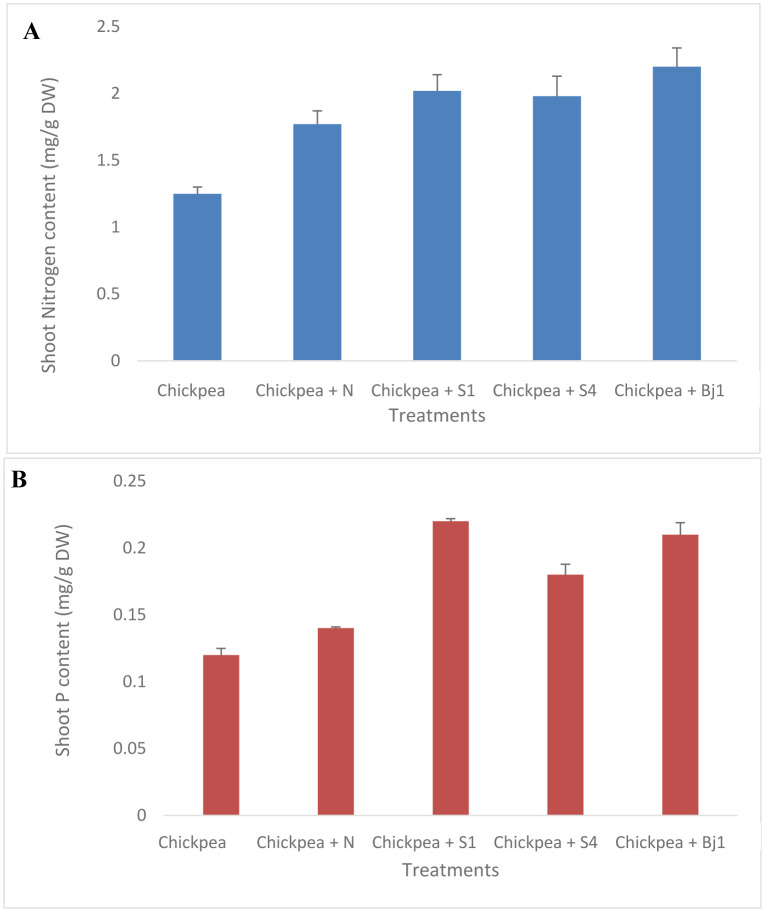
Nitrogen (A) and phosphorus contents (B) in chickpea shoots after inoculation with bacterial strains. Values are given as the means ± standard error (SE) of 10 replicates (n =10).

Analysis of the nutrients in chickpea shoots revealed that single inoculation using Strain 4, Strain 1, and *Mesorhizobium* Bj1 significantly increased N content by 58%, 68%, and 76%, respectively. P content was enhanced by 50% to 83% depending on the bacterial strains used (Figure 3).

### 4.4. In vivo assessment of biological control potential

Before using these bacteria in biological control tests, we checked their compatibility with the one *Mesorhizobium* strain Bj1 that was described as being specifically efficient for chickpeas. The mutually overlapping growth on yeast mannitol agar plates was identified as a beneficial pairwise relationship between the microorganisms. Every antagonist displayed a positive interaction with *Mesorhizobium*, demonstrating their mutually beneficial impact on plant growth.

**Figure 4.**
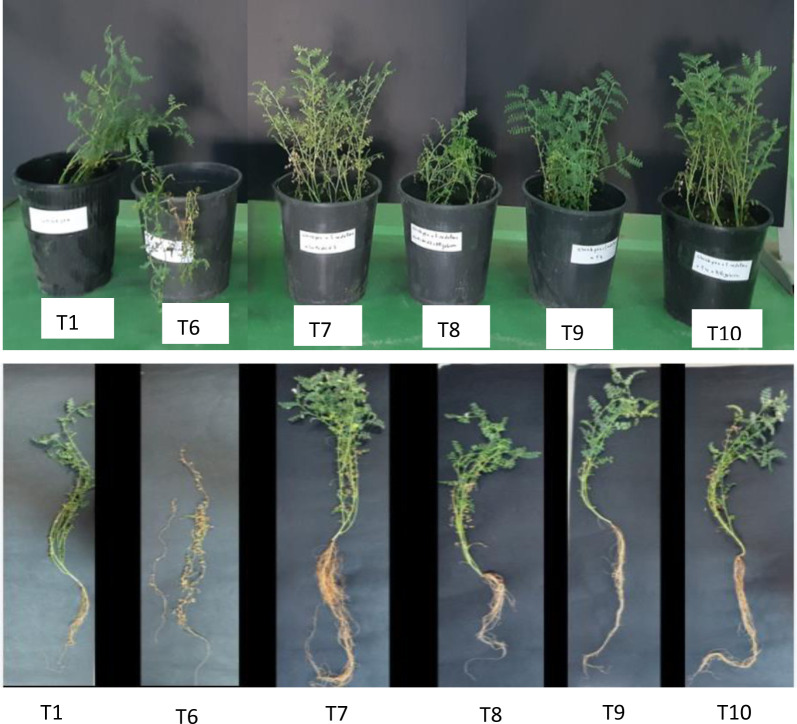
Disease severity in chickpea under growth room conditions. T1, Control ; T2, *F. redolens* (infected plants); T3, *F. redolens* + Strain 1; T4, *F. redolens* + Strain 1 + Bj1; T5, *F. redolens* + Strain 4; T6, *F. redolens* + Strain 4 + Bj1.

*F. redolens* significantly affected the growth of chickpea plants, and following infection with the fungus, there was a reduction in the dry biomass of the aerial parts of around 25% with a disease incidence of 75% (Tables 4 and 5 and Figure 4).

**Table 4.** Effects of two bacterial strains (S1, *Streptomyces diasticus* subp. *diasticus*; S4, *Bacillus subtilis*) and their combination with *Mesorhizobium* Bj1 on the biological control of chickpea wilt in pots.

	Treatments	Shoot length (cm)	Shoot dry weight (g)	Root length (cm)	Root dry weight (g)
T1	Chickpea	27.15 ± 4.25^b^	0.78 ± 0.09^bc^	42.11 ± 5.24^d^	0.58 ± 0.08^b^
T6	Chickpea + *F. redolens*	21.18 ± 3.47^a^	0.59 ± 0.07^a^	34.93 ± 3.89^a^	0,44 ± 0.1^a^
T7	Chickpea + *F. redolens* + S1	29.40 ± 3.25^d^	0.75 ± 0.08^bc^	39.05 ± 2.89^c^	0,60 ± 0.07^b^
T8	Chickpea + *F. redolens* + S4	27.10 ± 4.21^b^	0.71 ± 0.09^b^	37.17 ± 3.44^b^	0.48 ± 0.08^a^
T9	*F. Redolens* + S1 + Bj1	28.15 ± 2.85^c^	0.84 ± 0.1^c^	45.20 ± 4.11^e^	0.64 ± 0.08^c^
T10	*F. Redolens* + S4+ Bj1	28.70 ± 3.21^c^	0.72 ± 0.11^b^	43.70 ± 3.11^d^	0.62 ± 0.09^bc^

Results are expressed as the means (±SD) of 10 repetitions. Different letters indicate significance differences at the 5% level of probability using Duncan's multiple range tests.

**Table 5.** Disease severity index.

Treatments	Disease severity index of shoot	Disease severity index of root
Chickpea	0****	0****
Chickpea + *F. redolens*	2.75 ± 1.03	3 ± 0.9
Chickpea + *F. redolens* + S1	1.09 ± 0.3****	1 ± 0****
Chickpea + *F. redolens* + S1 + Bj1	2.1 ± 0.3*	1.9 ± 0.5****
Chickpea + *F. redolens* + S4	1.6 ± 0.5****	2 ± 0****
Chickpea + *F. redolens* + S4 + Bj1	1.36 ± 0.5****	1.81 ± 0.4****

The results are the mean (±SD) of 10 independent experiments. The statistical significance was determined using a two-way ANOVA complemented by Dunnett's multiple comparison test in comparison with the control (chickpea + *F.redolens*). Significance levels are as follows: * for *p* < 0.05, ** for *p* < 0.01, *** for *p* < 0.001, and **** for *p* < 0.0001.

Analysis of the results showed that two bacterial strains (Strains 1 and 4) significantly suppressed the disease's severity when inoculated separately (Tables 3 and 4 and Figure 4). For chickpea inoculated with *Streptomyces diastaticus* (S1) or *B. subtilis* (S4), no signs of necrosis were observed in the roots. Moreover, the co-inoculation of chickpea plants with Strain S1 and *Mesorhizobium* Bj1 was the best treatment for shoot growth (length and dry weight) compared with the control. The pathogen reduced the mean shoot dry weight by 25% (Figure 4).

## 5. Discussion

The use of microbial technology in the treatment of plant diseases has received considerable attention in recent years. In particular, the interaction of PGPRs with plants under biotic or abiotic stress is becoming increasingly important, with the aim of improving crop protection and increasing agricultural production. These biological control approaches are environmentally friendly, increasingly popular, reliable, and long-lasting. Improving plant growth by PGPRs is one of the outstanding properties of these indigenous microorganisms. The ability to improve plant growth and reduce plant disease is determined by the interaction between host plant and PGPRs [26]. PGPRs improve plant growth and health through both direct and indirect mechanisms that overcome diseases.

Out of 26 isolates tested against fungal strains in duplicate culture under *in vitro* conditions, seven isolates showed antagonistic potential against three to of the five fungal strains tested. However, variation in inhibition potential was observed even though the inoculum load was the same for all isolates. Among the positive antagonists, Isolates S2, S3, S4, S6, and S7 inhibited the five fungi tested, whereas Strain S1 inhibited four fungal strains and Strain S5 inhibited the growth of only three fungi. Variation in the inhibition effects was noted for several bacterial isolates and fungal strains. These inhibitions ranged from 40% to 80%. Similar results recorded by several studies [9],[27],[28] reported that 14 out of 96 *Pseudomonas* isolates from chickpea rhizosphere were highly antagonistic to a phytopathogenic fungus. The growth inhibition of fungi may be due to a fungistatic effect or could be attributed to the secretion of inhibitory metabolites by the antagonists.

In the present study, according to biochemical and molecular characterization, the isolated antagonistic bacteria belong to the families *Bacillaceae* and *Pseudomonadaceae*. These promising bacteria were further characterized biochemically and were found to be positive for one or more processes such as cellulosic activity, ammonia, siderophore, and IAA production. Three isolates were positive for cellulase production, which is consistent with available reports that biocontrol agents produce lytic enzymes and cellulase to limit pathogen invasion in plants [29]. Cellulase catalyzes the hydrolysis of 1,4-D-glycosidic bonds in cellulose, which contributes to the lysis of the pathogen's cell walls [30]. The hydrolytic enzymes produced by bacteria belonging to *Pseudomonas* sp. have been shown to contribute to disease suppression in chickpea and mung bean by inhibiting the growth of phytopathogenic fungi. In addition, the nodulation of legumes is improved by rhizobia [31]. All the identified antagonistic bacterial strains showed positive results for ammonia production. Ammonia is a recognized by-product of those rhizosphere bacteria that function as biocontrol agents [31]. The involvement of ammonia released by *Lysobacter capsici AZ78* in the inhibition of *Rhizoctonia solani* has been confirmed [32]. In the present study, the isolates tested positive for siderophore production. Competition for iron through the release of siderophores by antagonists has been reported to reduce the mycelial growth of the pathogenic fungus *B. cinerea*, which infects chickpea [33]. In another study, siderophore-producing rhizobacteria were shown to have a strong antagonistic effect against FOC [29]. Furthermore, four isolates produced IAA, a plant growth hormone with significant impacts on bacteria–plant interactions [34]. Additionally, these microbes were discovered to be effective in phosphate solubilization and nitrogen fixation, demonstrating the synthesis and release of different organic acids that cause nutrient solubilization. This is one of the ways that rhizobacteria promote plant growth, and also, by depriving the pathogen of vital nutrients and increasing the plants' access to nutrients [35].

Based on the antagonistic effect and the PGP traits, two bacterial strains (S1 and S4) were further subjected to *in vivo* studies to assess their potential effect on plant growth and for biological control of chickpea against *F. redolens* under pot conditions. When compared with the uninoculated control, co-inoculation of *S. diastaticus* subsp. *diastaticus* and *Mesorhizobium* sp. Bj1 resulted in significantly higher root and shoot dry weights. A similar result was observed for the co-inoculation of *B*. subtilus with *Mesorhizobium* sp. Bj1.

Earlier studies were conducted by Valverde et al. [36], which showed the impact of co-inoculation with the strains *Pseudomonas jessenii* PS06 and *Mesorhizobium ciceri* C-2/2 on the improvement of nodulation, growth, and seed yield of chickpea under greenhouse and field conditions. In a similar manner, inoculation of phosphate-solubilizing bacteria and *Rhizobium* in chickpea has been shown to improve yield, plant growth, nodulation, and nutrient intake [37],[38]. It is also known that phosphate-solubilizing bacteria enhance P uptake, which improves crop plants' growth and yield [39]. Studies combining PGPR and *Rhizobium*/*Bradyrhizobium* species in co-inoculation have been shown to boost the growth of several legumes, including lentil [40] and pigeonpea [41], as well as root and shoot biomass, nodule dry matter, nitrogenase activity, N_2_ fixation, and grain yield in chickpea [42]. Moreover, the combined effects of phosphate-solubilizing and N_2_-fixing rhizobacteria on plant growth, yield, grain protein, and nutrient uptake in chickpea plants have been documented by Verma et al [43]. When *S. diastaticus* subsp. *diastaticus* and *Mesorhizobium* strain Bj1 were co-inoculated, notable increases in root and shoot dry weight were observed. These increases can be attributed to both direct and indirect mechanisms that promote plant growth, including fixation of atmospheric N, siderophore production, solubilization of minerals like phosphate (P), phytohormone synthesis (a direct mechanism), suppression of phytopathogens, and the capacity of the latter to induce the systemic resistance of PGPRs (an indirect mechanism) compared with other strains.

Co-inoculation of beneficial bacteria can improve plants' resistance to fungal infections through several mechanisms. These bacteria, often beneficial microorganisms such as certain species of *Pseudomonas*, *Bacillus*, *Trichoderma*, and *Rhizobium*, can work in concert with plants to enhance their defense systems. Some researchers have shown that co-inoculation of legumes can reduce root diseases caused by *Fusarium* spp. and other pathogens and also has the potential to promote plant growth regardless of plant health [44]. The combination of *R. leguminosarum* + *A. chroococcum* + *P. fluorescens* was found to be more effective in controlling root rot and wilt diseases, increasing faba bean's growth parameters [45].

Many beneficial bacteria produce antimicrobial compounds (e.g., antibiotics and enzymes such as chitinases or siderophores) that directly inhibit the growth of fungal pathogens. For example, some strains of *Bacillus* produce metabolites that degrade fungal cell walls or disrupt their growth. A siderophore is a secondary metabolic product of microorganisms. It is secreted by bacteria, fungi, and other microorganisms and some plants in cases of iron deficiency. Studies have shown that siderophores can not only induce plant growth and significantly increase the biomass and length of roots and shoots, but also play an important role in controlling some plant pathogens [46]. Iron-chelating agents have been shown to enhance the activity of azole. Iron transporters, as iron-chelating agents, are potential new antibiotic and bacteriostatic agents. Antimicrobial substances produced in the noncontact inhibition pathway of bacteria include short-range lipopeptides and long-range volatiles. Lipopeptides are the most common antifungal agents in *Bacillus* spp. Small-molecule secondary metabolites are synthesized by the nonribosomal peptide synthetase (NRPS) pathway. *Bacillus* spp. are highly efficient producers of antibiotic molecules, and their inhibitory activity against plant pathogens through antibiosis is their best-known production mechanism [47]. *Bacillus* and *Pseudomonas* are known to have antifungal activity [48]. They produce many volatile compounds with antifungal activity [49]. Another study showed that *P. fluorescens* produces desferrioxamine, a hydroxamate siderophore, and its application improved oil content, nutrient uptake and plant growth in peanut [50]. Other studies have shown that the combination of α-Fe2O3 NP and *Trichoderma* can combat *Fusarium* wilt [51].

A significant reduction in the incidence of wilting was recorded in the roots and aerial parts of chickpea plants co-inoculated with PGPRs and *Mesorhizobium*. These results could be explained by the ability of bacterial strains to produce antifungal metabolites or the cumulative effect of different functional traits. Overall, our results showed that the combination of *Mesorhizobium* sp. Bj1 with the two PGPR bacteria (*S. diastaticus* subsp. *diastaticus* and *B. subtilis*) protected plants against *F. redolens* and improved growth parameters compared with plants attacked only by the fungus under controlled conditions. In summary, co-inoculation of plants with beneficial bacteria can improve their resistance to fungal infections by enhancing their immunity, competing with pathogens, producing antifungal compounds, and improving plant health and growth. This strategy can be a sustainable and effective alternative to chemical fungicides, contributing to healthier crops and reduced environmental impact. It would be interesting to confirm these observations under natural infestation conditions in different environments to adopt them as a strategy by farmers.

## 6. Conclusions

The results of the current study on co-inoculation with *S. diastaticus* subsp. *diastaticus* and *Mesorhizobium* sp. Bj1 strongly suggests a useful bioformulation to increase the growth of chickpea plants and to protect them against wilt. *B. subtilis* and *Mesorhizobium* sp. Bj1 compared with the treatment with *Mesorhizobium* sp. Bj1 by itself and the uninoculated control demonstrated that growth was also increased. On the other hand, the compatibility of the various bacteria and media components must be considered in bioformulations that combine different strains, nutrients, metabolites, and other natural products. In addition, it is important to conduct multi-site trials under agricultural conditions to confirm the results obtained under controlled conditions and to have more detailed information on the PGPRs and the biocontrol effects of the selected strains.
